# Preclinical Pharmacokinetic Studies of the Tritium Labelled D-Enantiomeric Peptide D3 Developed for the Treatment of Alzheimer´s Disease

**DOI:** 10.1371/journal.pone.0128553

**Published:** 2015-06-05

**Authors:** Nan Jiang, Leonie H. E. Leithold, Julia Post, Tamar Ziehm, Jörg Mauler, Lothar Gremer, Markus Cremer, Elena Schartmann, N. Jon Shah, Janine Kutzsche, Karl-Josef Langen, Jörg Breitkreutz, Dieter Willbold, Antje Willuweit

**Affiliations:** 1 Structural Biochemistry, Institute of Complex Systems (ICS-6), Forschungszentrum Jülich GmbH, Jülich, Germany; 2 Medical Imaging Physics, Institute of Neuroscience and Medicine (INM-4), Forschungszentrum Jülich GmbH, Jülich, Germany; 3 Structural and functional organisation of the brain, Institute of Neuroscience and Medicine (INM-1), Forschungszentrum Jülich GmbH, Jülich, Germany; 4 Department of Nuclear Medicine, Universitätsklinikum der RWTH Aachen, Aachen, Germany; 5 Institute of Pharmaceutics and Biopharmaceutics, Heinrich-Heine-Universität Düsseldorf, Düsseldorf, Germany; 6 Institut für Physikalische Biologie, Heinrich-Heine-Universität Düsseldorf, Düsseldorf, Germany; Cleveland Clinic Foundation, UNITED STATES

## Abstract

Targeting toxic amyloid beta (Aβ) oligomers is currently a very attractive drug development strategy for treatment of Alzheimer´s disease. Using mirror-image phage display against Aβ1-42, we have previously identified the fully D-enantiomeric peptide D3, which is able to eliminate Aβ oligomers and has proven therapeutic potential in transgenic Alzheimer´s disease animal models. However, there is little information on the pharmacokinetic behaviour of D-enantiomeric peptides in general. Therefore, we conducted experiments with the tritium labelled D-peptide D3 (^3^H-D3) in mice with different administration routes to study its distribution in liver, kidney, brain, plasma and gastrointestinal tract, as well as its bioavailability by i.p. and p.o. administration. In addition, we investigated the metabolic stability in liver microsomes, mouse plasma, brain, liver and kidney homogenates, and estimated the plasma protein binding. Based on its high stability and long biological half-life, our pharmacokinetic results support the therapeutic potential of D-peptides in general, with D3 being a new promising drug candidate for Alzheimer´s disease treatment.

## Introduction

After the initial description by Alois Alzheimer in 1906 [1], Alzheimer’s disease (AD), a progressive neurodegenerative disorder, has become nowadays the most common form (60–80%) of dementia [2]. According to the World Alzheimer Report 2014, nearly 36 million people worldwide are suffering from AD or related dementia. Even after years of intensive investigation and research, it is still an incurable disease [3]. Current treatments are only supportive against some of its symptoms. Clinical duration of AD varies from one to 25 years, typically eight to ten years [4].

Amyloid beta (Aβ) is produced by sequential cleavage of a type I integral transmembrane protein, called amyloid precursor protein (APP) by β- and γ-secretases. Variable lengths of Aβ isomers differing at the C-terminus are produced due to imprecise cleavage by γ-secretase [5, 6]. The most abundant isomers are Aβ1–40 (approximately 80–90%) and Aβ1–42 (approximately 5–10%). Aβ1–42 is more hydrophobic and fibrillogenic, and therefore the main component of Aβ plaques in the brain of AD patients [7]. It also aggregates readily into oligomers, which are considered to be the most toxic form of Aβ [8–10].

In recent years, many substances have been developed targeting Aβ production and clearance [11], including peptide-based drugs [12, 13]. In spite of the many advantages of peptide drugs, for example high specificity and low toxicity, their short half-life time *in vivo* due to rapid degradation by proteases, and low bioavailability by oral administration, restrict their clinical usage. In comparison to naturally occurring L-form peptides, peptides derived from partial D-amino acid substitutions or D-enantiomeric peptides, which are composed entirely of D-amino acids, have advantages over L-enantiomers. Because of the stereoisomeric selectivity of proteolytic enzymes they are less prone to proteolysis, therefore longer half-lives and higher bioavailability after oral administration are to be expected [14–16]. Furthermore, they are less or even not immunogenic at all [13].

The fully D-enantiomeric peptide D3, which was identified by mirror-image phage display [17, 18] for binding to Aβ (1–42), has been shown to have interesting properties. D3 inhibits Aβ fibril formation and eliminates Aβ-oligomers *in vitro*. *Ex vivo*, D3 has been shown to specifically bind to amyloid plaques in transgenic mice [19]. *In vivo*, D3 was able to reduce plaque load and inflammation markers in the brains of treated transgenic mice, as well as improve their cognition even after oral administration [20–23]. Here we investigate the pharmacokinetic properties of D3 in mice.

We present the first comprehensive preclinical pharmacokinetic study of a peptide consisting solely of D-enantiomeric amino acid residues in general and in particular for such a D-peptide developed for the treatment of Alzheimer’s disease.

## Materials and Methods

### Materials

^3^H-D3 (rprtr-(4,5-^3^H-Leu)-hthrnr) and its L-form enantiomer ^3^H-(L)-D3 (RPRTR-(4,5-^3^H-Leu)-HTHRNR) were purchased from Quotient Bioresearch (Radiochemicals) Ltd. (Cardiff, United Kingdom) with 10–100 Ci/mmol, 1 mCi/ml and purity >95%.

All chemicals were supplied by Fluka Chemie AG (Buchs, Switzerland), Merck (Darmstadt, Germany), AppliChem (Darmstadt, Germany) and VWR (Darmstadt, Germany) in research grade. Micro-osmotic pumps (model 1007D) were purchased from Alzet DURECT Corporation, (Cupertino, CA, USA).

### Animals

Male C57Bl/6 mice (Charles River, Sulzfeld Germany) with an average age of 13 weeks and body weight of 28.5 g were used in this study. For micro-osmotic pump i.p. implantation experiment, 19 months old mice were used with average body weight of 34 g. The mice were hosted in the animal facility of the Forschungszentrum Juelich under standard housing conditions with free access to food and water for at least 2 weeks before experiment. All animal experiments were approved by the Animal Protection Committee of the local government (LANUV (Landesamt für Natur, Umwelt und Verbraucherschutz), North-Rhine-Westphalia, Germany, AZ84-02.04.2011. A359 and AZ84-02.04.2011. A356) according to the Deutsche Tierschutzgesetz). All sections of this study adhere to the ARRIVE Guidelines for reporting animal research [24]. A completed ARRIVE guidelines checklist was included in Supporting Information (S1 File).

### Pharmacokinetic studies

Mice were administered with 100 μl radioactive working solution consisting of 5 μCi ^3^H-D3 in 5 μl with 95 μl buffer (0.1 M phosphate buffer, pH 8) as a single bolus dose either i.v. (tail vein), i.p. or p.o. (gavaging). In order to achieve the desired total D3 concentration, non-radioactive D3 was added to a concentration of 1 mg/ml (i.v.) or 3 mg/ml (i.p. and p.o.). Doses were selected from previous tolerability studies and were not causing any adverse effects. I.v. injections and i.p micro-osmotic pump implantations were performed under anaesthesia with ketamine/medetomidine per i.p. administration. Antisedan was administered s.c. to reverse the anaesthesia directly after the intervention, which took about 10 min. Sampling times were chosen depending on the route of administration (i.p.: 10, 20, 30, 60, 120, 240, 360, 1440 and 2880 min.; p.o.: 10, 20, 30, 60, 120, 240, 360, 1080, 1440, 2880 and 4320 min.; i.v.: 3, 5, 10, 15, 30, 60, 240, 1440 and 2880 min.; 3 animals per time point). For i.p. micro-osmotic pump implantation, delivery dose of pumps was set to 5 μCi ^3^H-D3 plus 0.3 mg non-radioactive D3 per 24 hours per mouse. Sampling times were 2, 4 and 6 days after implantation (3 mice per time point).

Upon sampling time, blood was drawn per heart puncture under isoflurane anaesthesia and heparinized plasma was isolated. A small piece of liver (approx. 0.2 g), the left kidney and the right brain hemisphere were sampled. To study the gastrointestinal absorption and elimination by p.o. administration, mice were fasted 18 hours before the experiment and their complete gastrointestinal tracts were prepared. Small intestine was dissected into 4 equal parts and marked from oral to aboral as 1 to 4, respectively. Organ samples were weighted and homogenized in homogenizer tubes (Precellys Ceramic Kit 1.4 mm, Precellys 24, Bertin technologies SAS, Montigny le Bretonneux, France) with 500 μl PBS. 10 ml scintillation cocktail (Ultima Gold XR, PerkinElmer, Waltham, Massachusetts, USA) was added to 100 μl of each organ homogenate or plasma (diluted 1:1 with PBS) and mixed well. Disintegrations per unit time (dpm) were obtained in triplicates with a liquid scintillation counter (Packard Tri-Carb 2100TR Liquid Scintillation Analyser, PerkinElmer, Waltham, MA, USA). Blank values of each sample were obtained by omitting radioactive substance following the same protocol.

Radioactivity counted in each sample was adjusted (subtraction of the blank value) and was expressed as percentage injected dose per gram tissue or millilitre plasma (%ID/g or %ID/ml), or as milligram of total D3 per gram tissue or millilitre plasma (mg/g or mg/ml).

### Pharmacokinetic analysis

Pharmacokinetic parameters were calculated with non-compartmental analysis using Phoenix WinNonlin, version 6.3 (Pharsight Corp., St. Louis, USA). Mean D3 concentrations per time point were used to calculate the PK parameters (model type: plasma (200–202); calculation method: linear trapezoidal linear interpolation; dose options: “IV Bolus” for i.v. or “Extravascular” for i.p. and p.o. administration). The same model setting was used to estimate pharmacokinetic parameters of brain. For i.v. administration, plasma concentration at time zero (C0) was back extrapolated with a log-linear regression of the first two observed plasma concentrations, while brain C0 was set to be zero. For the i.p. and p.o. administrations, all concentrations at time zero were set to be zero.

The last three to five observed mean plasma concentrations were used to estimate the first order rate constant in the terminal elimination phase (Lambda_z) based on the largest adjusted square of the correlation coefficient (R^2^) of the log-linear regression lines. The area under the curve (AUC) from C0 extrapolated to infinity (AUC_C0-inf_) was calculated as the sum of AUC_C0-last_+(Clast/Lambda_z), calculated from the last determined concentration derived by Lambda_z, and AUC_C0-last_ representing the AUC from time point zero to the last observed concentration (Clast). Parameters that do not require Lambda_z were calculated for brain data: time of maximal observed concentration (Tmax), maximal observed concentration (Cmax), maximal observed concentration normalized to dose (Cmax/D), AUC_C0-last_ and mean residence time from the time of dosing to the last time point (MRT_C0-last_). Additional parameters requiring estimated Lambda_z were calculated for plasma data: Lambda_z, terminal half-life (HL_Lambda_z), AUC_C0-inf_, terminal volume of distribution (Vz), plasma clearance (Cl), MRT_C0-inf_ and volume of distribution at steady state (Vss). Absolute bioavailability of i.p. and p.o. administration was calculated with AUC_C0-inf_ by: F(bioavailability) = [AUC(non-iv)*Dose(iv)]/[AUC(iv)*Dose(non-iv)]*100.

To minimize the time dependence of brain-plasma ratio by bolus dosing, brain-plasma ratio was calculated from the areas under the brain and plasma concentration curves in the terminal elimination phase starting from 4 hours to infinity (brain_AUC_4h-inf_/plasma_AUC_4h-inf_).

### Plasma protein binding

Plasma protein binding was estimated by incubation of D3 with varying concentrations of protein using TRANSIL^XL^ binding kits (Sovicell GmbH, Leipzig, Germany). K_D_ values were determined by titrating a constant drug concentration against different concentrations of human serum albumin (HSA) and α_1_-acid glycoprotein (AGP). Experiments were performed as recommended for the kit. To obtain the desired D3 stock solution of 80 μM, non-radioactively labelled D3 was dissolved in PBS and 5% ^3^H-labelled D3 solution was added for detection purposes. A final concentration of 5 μM D3 was applied in the assay. After incubation and centrifugation 15 μl supernatant were taken and scintillation cocktail was added. This was done in triplicate. Radioactivity was then quantified using liquid scintillation counting. After measuring the disintegrations per minute (dpm) of the supernatant containing the unbound peptide, the D3 fraction bound to the titrated protein was calculated and plotted against the protein concentrations. The curves were fitted to the Michaelis Menten ligand binding equation (SigmaPlot 11.0, Systat Software, Inc., San Jose, California, USA) to obtain the K_D_. Mean and relative standard error (%) of multiple measurements are given (AGP n = 3, HSA n = 2).

For bioavailability determination, the unbound fraction of D3 (f_u_) was calculated using the equation below:

$$f_{u}=100*\frac{\frac{C_{D3}-K_{D}-C_{physiol}}{2}+\sqrt{K_{D}*C_{D3}+{\left(\frac{C_{D3}-K_{D}-C_{physiol}}{2}\right)}^{2}}}{C_{D3}}$$ (1)

For very low D3 concentrations in blood (C_D3_), Eq (1) can be simplified by Eq (2), where the unbound fraction of D3 can be calculated independently of the applied D3 concentration. Since this is true for our *in vivo* experiments we used Eq (2) for the total free fraction of D3, combining the binding of D3 to HSA and AGP. For calculation of the overall unbound fraction according to Eq (2), physiological concentrations (C_physiol_) of 0.65 mM HSA and 0.02 mM AGP were assumed.

$$f_{u,total}=100*\frac{1}{1+\frac{C_{physiolHSA}}{K_{D}HSA}+\frac{C_{physiolAGP}}{K_{D}AGP}}$$ (2)

### Calibration curves and internal standard

Calibration curves were prepared by adding a corresponding ^3^H-D3 dilution series with certain dpm range to plasma or organ homogenates in comparison to those diluted in PBS. The dpm ranges of each ^3^H-D3 dilution series were set to cover the measured dpm ranges of each sample (for plasma 400–40000; for brain 100–1200; for liver 3000–15000; for kidney 40000–400000). Plasma and organ homogenates obtained from C57Bl/6 mice were prepared following the same procedure as outlined above.

No differences were found comparing the calibration curves of ^3^H-D3 in organ homogenates or plasma to those in PBS. The measured dpm values of the internal standard with ^3^H-D3 in PBS matched closely the expected ones.

### Thin layer chromatography

In order to study the proteolytic stability of peptides in biological extracts, tritium labelled peptides were incubated with liver microsomes (pooled from mouse (CD-1), Sigma-Aldrich), freshly prepared mouse plasma or extracts of brain, liver and kidney at 37°C for different time periods (from 0 min to 2 days). 1 μCi (approx. 0.08–0.8 μg) radioactive labelled peptide was mixed with 1 μl microsomes stock solution, plasma or organ extracts, respectively (in great excess to peptide). Mixtures containing tritium-labelled peptides were applied onto HPTLC Silica Gel 60 plates (OMNILAB, Essen, Germany) for thin layer chromatography (TLC) with a mobile solvent (2-Butanol/Pyridine/Ammonia(28%)/Water(39/34/10/26)). After development, a phosphor imaging plate for ^3^H-autoradiography (FUJIFILM, Tokyo, Japan) was exposed to the TLC plates for 3 days. Images were acquired with a BAS reader and AIDA software (Raytest, Freiburg, Germany). Retardation factor (Rf) of each substance was defined as the ratio of the migration distance of the centre of a separated spot to the migration distance of the solvent front.

## Results

### Proteolytic stability of D3 in comparison to its L-enantiomer

**Fig 1:**
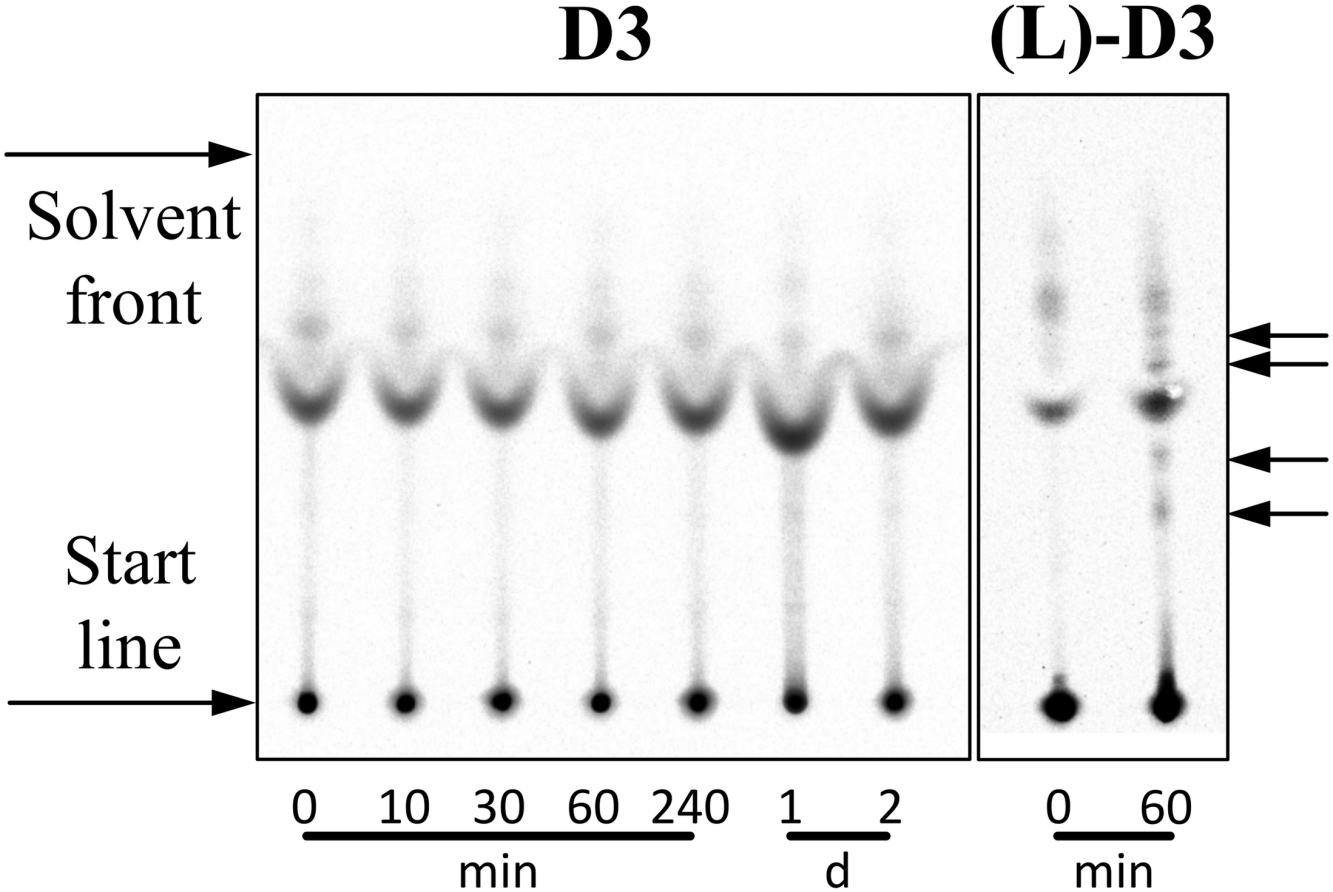
Autoradiogram demonstrating proteolytic stability of ^3^H labelled peptides in plasma. ^3^H-D3 was incubated with plasma for different times at 37°C and developed on TLC plates. For comparison, the exact enantiomer of D3, (L)-D3, was used in this stability assay. ^3^H-(L)-D3 was incubated with plasma for 0 and 60 min at 37°C. Please note that free ^3^H-(L)-D3 and free ^3^H-D3 are perfect enantiomers to each other and because the TLC material is not chiral, both compounds show identical Rf values. Additional bands in the 0 min lanes of ^3^H-(L)-D3 and ^3^H-D3 that arise from binding and co-migration of ^3^H-D3 and ^3^H-(L)-D3 to plasma components do not necessarily have identical Rf values in the 0 min lanes of ^3^H-(L)-D3 and ^3^H-D3, because some of the plasma components are enantiomers themselves. Therefore, any effect of degradation will lead to extra additional bands as compared to the 0 min lane of the very same compound. Obvious proteolytic degradation can be observed for ^3^H-(L)-D3 already after 60 min incubation with plasma leading to additionally appearing bands (black arrows) as compared to the 0 min lane ^3^H-(L)-D3. Additionally appearing bands as compared to 0 min incubation are not observed for ^3^H-D3 even after 2 days incubation.

Before meaningful pharmacokinetic studies could be performed with ^3^H-D3, it was essential to show that the D-peptide is stable under near *in vivo* conditions. First, we compared the stability of ^3^H-D3 with its exact enantiomer, ^3^H-(L)-D3 in plasma (Fig 1). ^3^H-(L)-D3 shows significant degradation already after 60 min incubation in plasma as concluded by the appearance of additional bands as compared to the mixture at 0 min on the TLC plate after detection by autoradiography. In contrast, ^3^H-D3 did not show any degradation products even after 2 d incubation in the same plasma preparation.

**Fig 2:**
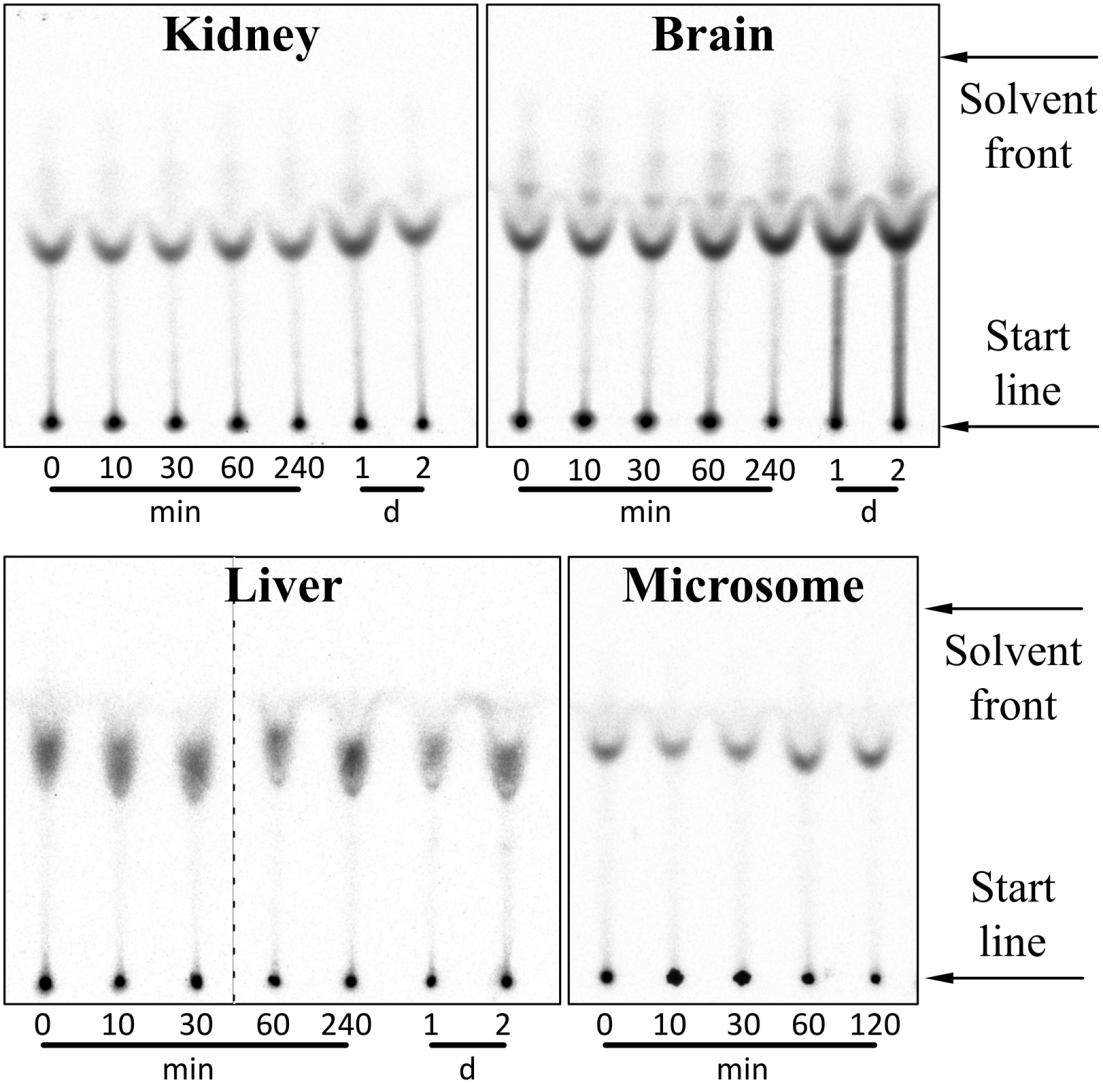
Autoradiogram demonstrating proteolytic stability of ^3^H labelled peptides in liver microsomes and organ homogenates. ^3^H-D3 was incubated with kidney, brain and liver homogenate for 0, 10, 30, 60, 240 min and 1, 2 days at 37°C and developed on TLC plates. For liver microsomes, the incubation time was 0, 10, 30, 60 and 120 min. Slight difference in Rf values of ^3^H-D3 in liver homogenate might be due to incompletely homogenized liver tissues, which was not observed after incubation with liver microsomes. (Two autoradiograms of liver homogenate were presented in one image and separated through a dashed line.) No obvious proteolytic degradation of D3 can be observed in all the organ homogenates with up to two days’ incubation.

More importantly, ^3^H-D3, was neither degraded after 2 h incubation in liver microsomes nor after 2 days incubation in homogenates of kidney, brain and liver as shown by TLC and detection by autoradiography (Fig 2). Microsomes were checked for proteolytic activity using l-peptide substrates.

**Fig 3:**
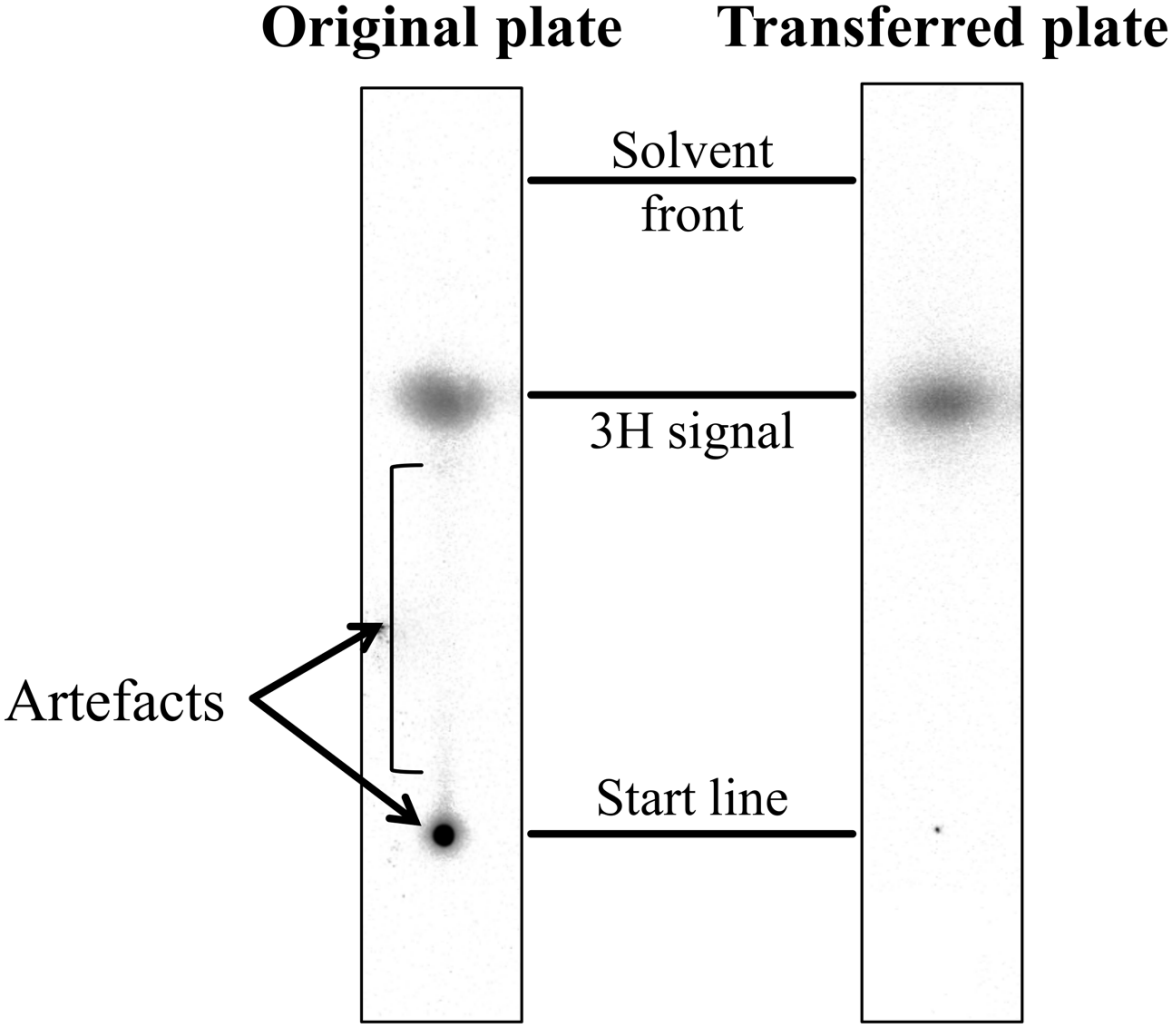
Plate-transfer of ^3^H-D3 in TLC matrices. A control experiment was performed by placing a new TLC plate to a freshly developed plate to transfer only the ^3^H-D3 within matrices. On the mirror image of the transferred plate, the ^3^H signals at the start points as well as the smears were obviously reduced, while the intensity of separated ^3^H-D3 did only change slightly. This result suggests that the observed artefacts arise from unspecific ^3^H-D3 binding to the glass surface.

Due to high but unspecific affinity of D3 and (L)-D3 to the TLC plate support material (glass), artefacts were observed at the starting points of the TLC as well as light smears originating thereof. To prove that these compounds were not located in the layer of the TLC matrices, a control experiment was performed by placing a new TLC plate to a freshly developed plate to transfer only the ^3^H-peptides within matrices, but not those on the glass surface support (Fig 3). Artefacts could thus be eliminated.

### Pharmacokinetics

**Fig 4:**
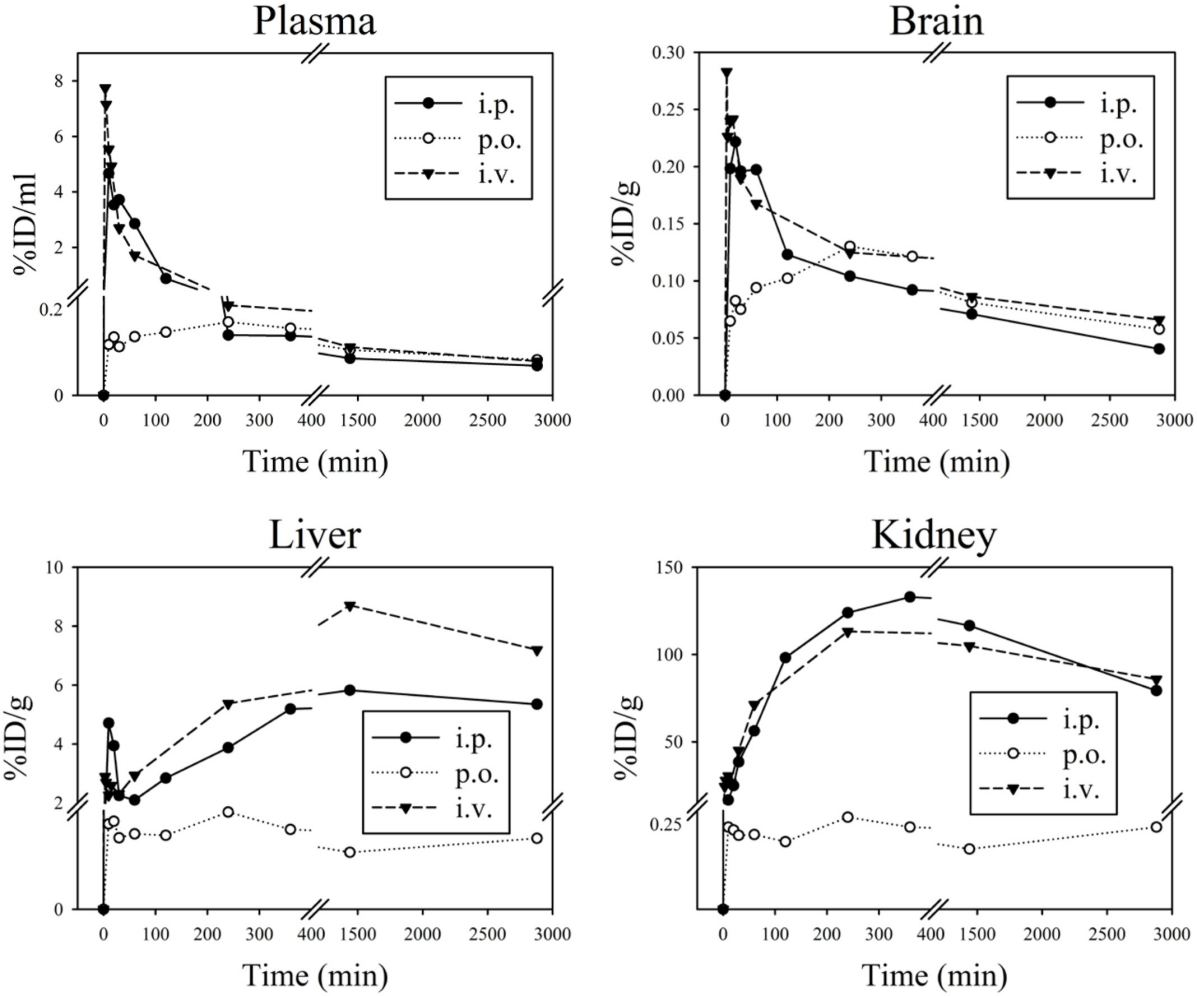
Mean pharmacokinetic profiles of ^3^H-D3 in organs and plasma after i.p., p.o. and i.v. administration. ^3^H-D3 (5 μCi) mixed with D3 in a total concentration of 3.5 mg/kg (i.v.) or 10.5 mg/kg (i.p. and p.o.) was applied per mouse. D3 concentrations are shown as percentage of injected dose per gram tissue or milliliter plasma (%ID/g or %ID/ml) dependent of time after administration. Mean values from 3 mice are shown.

Time dependent distribution of D3 in organs and plasma after different administration routes was analysed using tritium labelled D3 (^3^H-D3) as shown in Fig 4. The corresponding pharmacokinetic parameters calculated with non-compartmental analysis based on the absolute amount of administered D3 are shown in Tables 1 and 2.

**Table 1:** Pharmacokinetic parameters for D3 from noncompartmental analysis of plasma.

Parameter	Units	i.v. (3.5 mg/kg)	i.p. (10.5 mg/kg)	p.o. (10.5 mg/kg)
Tmax	min	3	10	240
Cmax	μg/ml	7.75	14	0.45
Cmax/D	μg/ml/mg	77.5	46.7	1.48
AUC_C0-last_	min*μg/ml	679	1763	1095
MRT_C0-last_	min	547	527	1718
Lambda_z	1/min	0.00036	0.00028	0.00028
HL_Lambda_z	min	1907	2471	2439
AUC_C0-inf_	min*μg/ml	869	2404	1521
MRT_C0-inf_	min	1658	2104	3430
Vz	ml	317	445	684
Cl	ml/min	0.115	N.A.	N.A.
Vss	ml	190	N.A.	N.A.
Bioavailability	%	N.A.	92.2	58.3

N.A.: Parameters not applicable for this administration route. For abbreviations see methods section.

**Table 2:** Pharmacokinetic parameters for D3 from noncompartmental analysis of brain.

Parameter	Units	i.v. (3.5 mg/kg)	i.p. (10.5 mg/kg)	p.o. (10.5 mg/kg)
Tmax	min	3	20	240
Cmax	μg/g	0.283	0.665	0.390
Cmax/D	μg/g/mg	2.83	2.22	1.30
AUC_C0-last_	min*μg/g	275	643	935
MRT_C0-last_	min	1173	1108	1693

For abbreviations see methods section.

**Fig 5:**
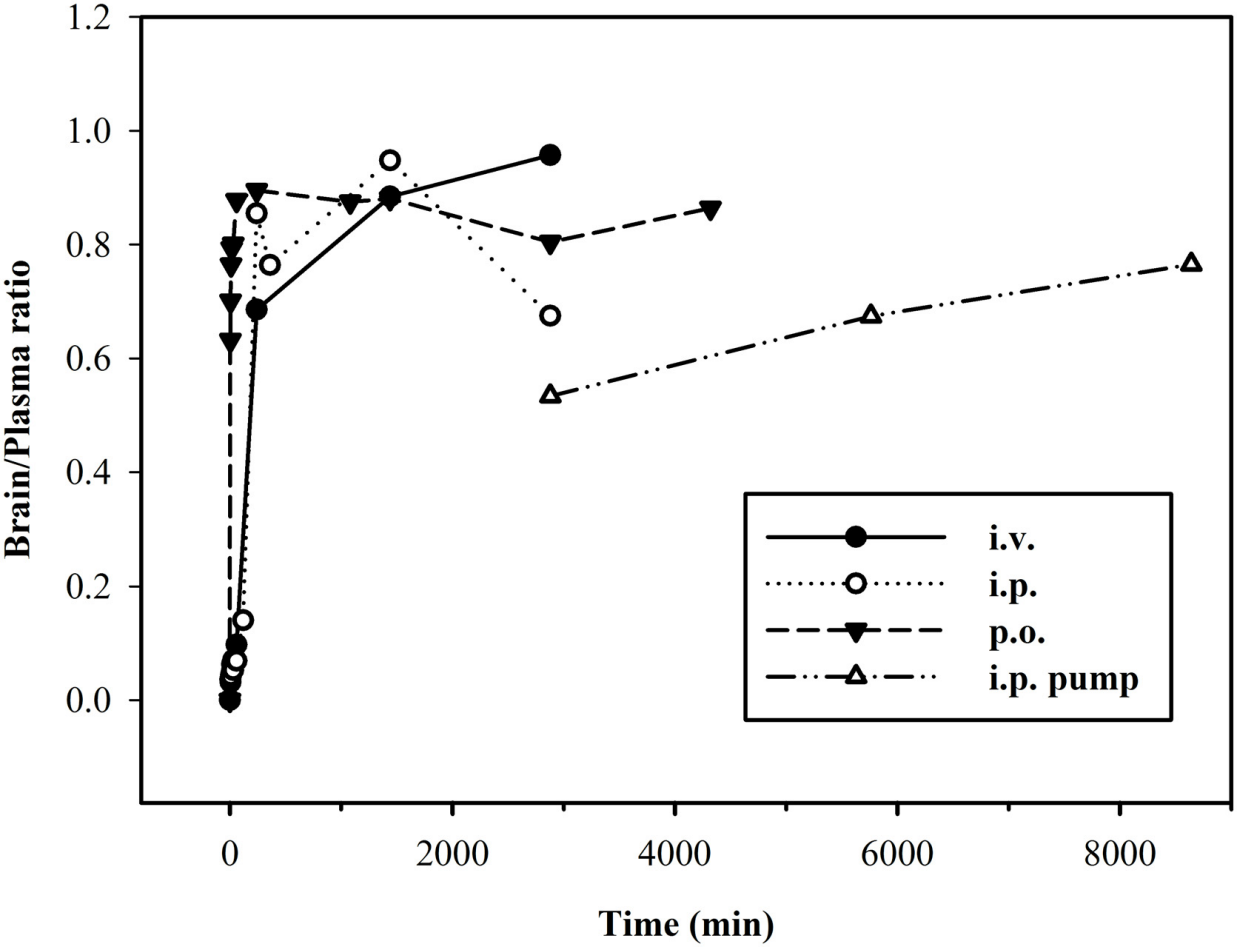
Temporal distribution of brain/plasma ratio of ^3^H-D3 after different administration routes. Following bolus dose administration, low brain/plasma ratios were found at the starting time points. After 4 hours, the ratios reached relative high values and varied between 0.7–1.0. Upon i.p. pump implantation the ratio increased constantly with time.

After i.v. and i.p. administration, pharmacokinetic curves showed similar patterns with highest concentration of tritium per gram tissue found in kidney, followed by liver and plasma. However, after oral administration ^3^H-D3 concentrations measured in kidney and liver did not exceed concentrations in plasma (Fig 4). Plasma Cmax/D after i.v. administration reached 78 μg/ml/mg at Tmax 3 min (the first sampling time point), while after i.p. and p.o. administration plasma Cmax/D were 47 μg/ml/mg at 10 min and 1.5 μg/ml/mg at 240 min (Table 1). In brain, the Cmax/D and their corresponding Tmax values for i.v., i.p. and p.o. administration were 2.8, 2.2 and 1.3 μg/ml/mg at 3, 20 and 240 min, respectively (Table 2). However, after 4 hours concentrations in brain reached similar concentrations irrespectively of the administration route (Fig 4). Although plasma concentrations after p.o. administration appeared to be very low in comparison to i.v. and i.p. administration, comparable concentrations of ^3^H-D3 were found in the brain resulting in high brain/plasma ratio after 4 h (Fig 5).

4 hours after a ^3^H-D3 bolus dose, brain/plasma ratio of all administration routes reached a plateau between 0.7 and 1.0 (Fig 5). To minimize the time dependence of brain/plasma ratio, the absolute ratios were calculated from the area under the brain and plasma concentration curves from 4 hours to infinity (brain_AUC_4h-inf_/plasma_AUC_4h-inf_) with 1.07 for i.v., 0.69 for i.p., and 0.85 for p.o. administration.

After bolus administration, D3 showed relatively long elimination half-lives in plasma of 31.8 h, 41.2 h and 40.7 h after i.v., i.p. and p.o. administration, respectively. Plasma clearance was 0.12 ml/min after i.v. administration. Apparent volumes of distribution were different among i.v., i.p. and p.o. administration with 316, 444 and 684 ml, respectively (Table 1).

**Fig 6:**
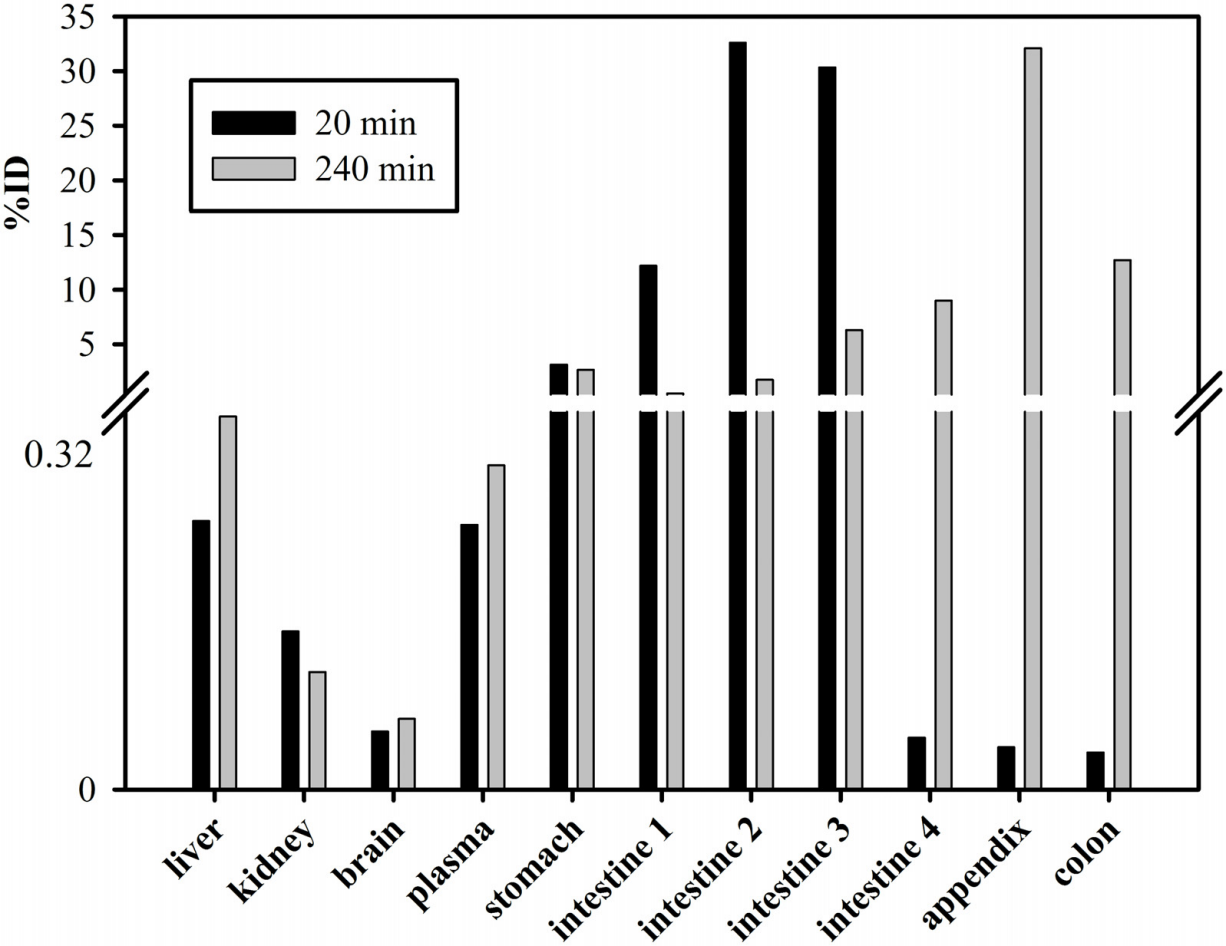
Distribution of ^3^H-D3 after p.o. administration in organs and plasma. 20 min after gavaging of 100 μl, 5 μCi ^3^H-D3 with a total D3 concentration of 10.5 mg/kg, most of the radioactivity was located in the middle of small intestine (intestine 2 and 3); 4 hours later, it spread to the lower intestinal tract. Of note is the high concentration of D3 observed in the appendix. At this time point, D3 could already be detected in feces. In comparison to the gastrointestinal tract, the amount of D3 in other organs or plasma after p.o. administration was very low.

Absolute bioavailability was high with 92.2% after i.p. administration and 58.3% after p.o. administration (Table 1). When studying gastrointestinal distribution of D3 after p.o. administration (Fig 6), most of the radioactivity was found in the lower intestinal tract after 4 hours, which suggested that the majority of D3 did not enter the system circulation within 4 hours. Still, the AUC of D3 in brain after p.o. administration was comparable to those after i.p. and i.v. administration (Table 2).

**Fig 7:**
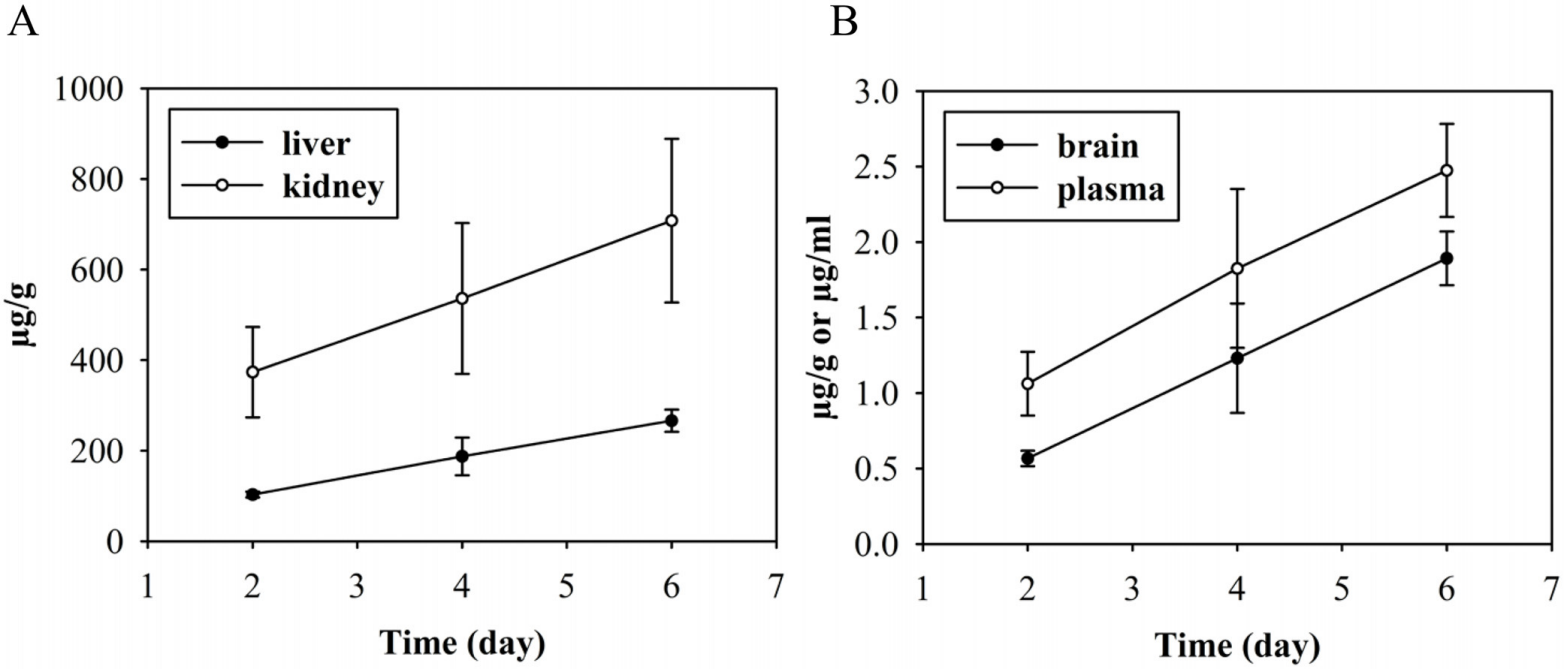
Concentration of ^3^H-D3 in kidney, liver, brain and plasma administered via i.p. implanted osmotic pump. Alzet mini pumps with a delivery rate of 0.3 mg D3 (plus 5 μCi ^3^H-D3) per 24 hours were implanted i.p. and organs were sampled after 2 to 6 days. Similar to bolus i.p. administration, more ^3^H-D3 was found in kidney than in liver (A), whereas D3 concentrations in plasma and brain were considerably lower (B). The concentration of D3 was increasing linearly over time suggesting that the saturation concentration in the respective organs and plasma was not reached by 6 days of continuous dosing.

We were also interested in answering the question, whether continuous dosing over several days using an i.p. implanted osmotic pump is showing specific effects in D3 distribution. We found linearly increasing D3 concentrations in plasma and all tested organs over 6 days (Fig 7). Although D3 highly accumulated in liver and kidney at day 6, the mice did not show any obvious signs of intoxication. The brain/plasma ratio increased with time from 0.53 at day 2 to 0.77 at day 6.

### Plasma protein binding of D3

**Fig 8:**
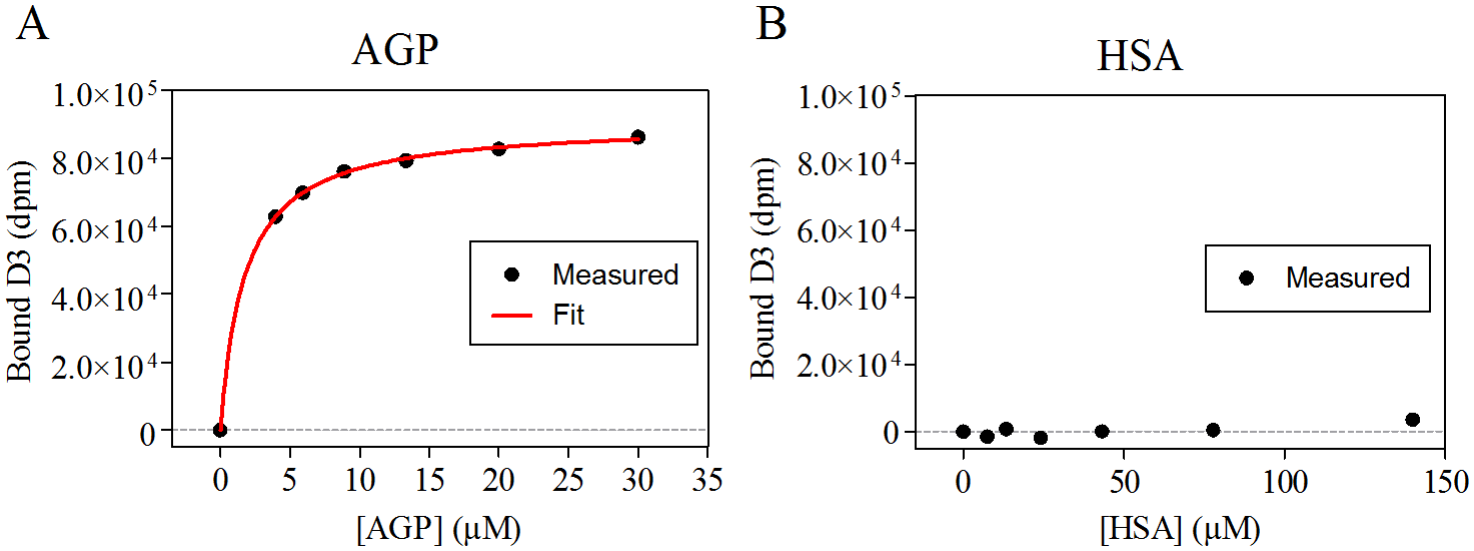
The bound D3 (in dpm) over the protein concentration as determined using the TRANSIL^XL^ kits. Each sample contained 5 μM D3 added to varying concentrations of AGP or HSA. (A) AGP fitted to the Michaelis Menten equation (red). (B) The binding of D3 to HSA was below the detection limit of the kit (K_D_ > 1.4 mM).

To estimate the free fraction of D3 in plasma *in vivo* (f_u,total_), D3 was incubated with human serum albumin (HSA) and α_1_-acid glycoprotein (AGP) in an *in vitro* assay (Fig 8). The plasma protein binding assay for AGP resulted in a K_D_ of 1.8 μM ± 7.9%. Assuming a D3 concentration in blood of 0.1 μM (C_D3_, measured 4 h after i.p. injection) calculation of binding to AGP according to Eq (1) predicts a free fraction of 8.3%. For HSA, the K_D_ was above the detection limit of the kit (> 1.4 mM) indicating very low affinity of D3 to HSA. Nevertheless, calculation of the free fraction with an assumed K_D_ of 1.4 mM resulted in 68.3% free D3. Taken together, using Eq (2), the estimated free fraction of D3 in plasma was calculated to be approximately 8%.

## Discussion

In the current study we have analysed the distribution of the D-enantiomeric peptide D3 after single intravenous, intraperitoneal and per oral administration, as well as continuous dosing via intraperitoneally implanted osmotic pumps. To the best of our knowledge, this is the first report of a comprehensive pharmacokinetic study of a peptide consisting solely of D-enantiomeric amino acid residues in rodents demonstrating excellent proteolytic stability, long plasma half-life and very high oral bioavailability.

D3 showed high proteolytic resistance exactly as it was shown *in vitro* previously with other all-D-peptides [14–16]. Thanks to this stability, metabolites can be neglected and the measured ^3^H radioactivity represents the concentration of D3 after administration *in vivo*.

Estimated terminal plasma half-lives of D3 were between 32 and 41 h and were thus much higher than those reported for L-enantiomeric peptides which are typically only a few minutes [25]. Four hours after administration, irrespective of the administration routes, the temporal distribution of D3 in brain closely followed that in plasma resulting in brain/plasma ratios between 0.7 and 1.0 (Fig 5). While substances with a brain/plasma ratio larger than 0.3 are considered to have sufficient access to the central nervous system [26], our results suggest that D3 efficiently overcomes the blood-brain barrier.

Interestingly, by p.o. administration of D3, in spite of only a small rate of D3 being absorbed via the enteric tract, the bioavailability was 58.3% (Table 1), which is relatively high in comparison to that of L-peptide drugs, which were described to be less than 1% without delivery enhancement [27–30]. This finding can be explained by slow oral absorption of D3 and particularly long terminal half-life in plasma resulting in high AUC-values after p.o. administration (Table 1). Low concentrations of D3 as found in kidney and liver after p.o. administration are desirable because this lowers the risk of possible intoxication of important organs. With absorption enhancers and a more suitable formulation of D3, even higher oral bioavailabilities seem to be feasible. Due to the observed high stability of D3 against proteolysis under biological conditions and its hydrophilic properties, elimination via biliary excretion (without re-absorption) and renal clearance in unchanged form could be expected.

Estimated volumes of distribution were 11.1 (i.v.), 15.6 (i.p.) and 24.0 l/kg (p.o.), respectively considering the body weight of the mice (28.5 g in average). The total body water in C57Bl/6 mice is approximately 0.6 l/kg [31], suggesting a distribution of D3 beyond the body fluid and some uptake in peripheral tissues.

Plasma volume of distribution at steady state was also high with 191 ml and 6.69 l/kg considering the body weight of the mice and the fraction of unbound D3 in plasma was predicted to be around 8%. High volume of distribution promotes low plasma clearance, which in our study was approximately between 0.12–0.19 ml/min observed in all routes of administration.

In summary, the current study demonstrates high proteolytic stability for the D-enantiomeric peptide D3. Furthermore, D3 enters the brain very efficiently and shows high oral bioavailability. The terminal half-life in mice after p.o. administration was approximately 41 hours with a brain/plasma ratio between 0.7 and 1.0, and a bioavailability of about 60%.

In our previous studies, D3 already proved to be therapeutically active in reversing cognitive deficits and amyloid plaque load *in vivo*. Given its high oral bioavailability, suitably formulated D3 with multiple dosing might be a promising drug candidate against Alzheimer’s disease.

## Supporting Information

S1 File ARRIVE Checklist. Completed “The ARRIVE Guidelines Checklist” for reporting animal data in this manuscript.
(DOCX)
