# Supplementary material for: Preclinical Pharmacokinetic Studies of the Tritium Labelled D-Enantiomeric Peptide D3 Developed for the Treatment of Alzheimer´s Disease
Source: PLoS One. 2015 Jun 5;10(6):e0128553. doi: 10.1371/journal.pone.0128553 (PMC4457900; doi:10.1371/journal.pone.0128553)

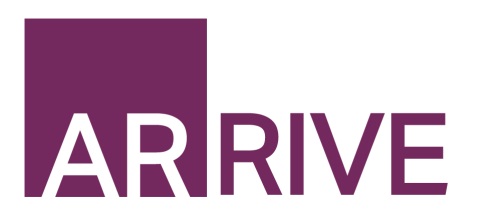


The ARRIVE Guidelines Checklist

Animal Research: Reporting In Vivo Experiments

Carol Kilkenny^1^, William J Browne^2^, Innes C Cuthill^3^, Michael Emerson^4^ and Douglas G Altman^5^

*^1^The National Centre for the Replacement, Refinement and Reduction of Animals in Research, London, UK, ^2^School of Veterinary Science, University of Bristol, Bristol, UK, ^3^School of Biological Sciences, University of Bristol, Bristol, UK, ^4^National Heart and Lung Institute, Imperial College London, UK, ^5^Centre for Statistics in Medicine, University of Oxford, Oxford, UK.*

|  | | ITEM | RECOMMENDATION | Section/ Paragraph |
| --- | --- | --- | --- | --- |
| 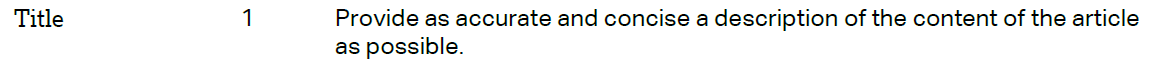 | | | Title |  |
| 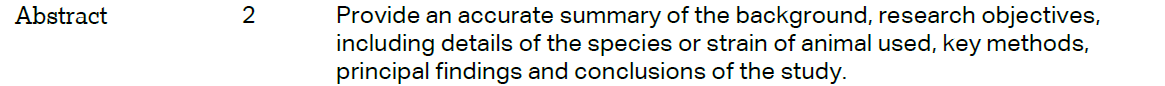 | | | Abstract |  |
| INTRODUCTION | | |  |  |
| 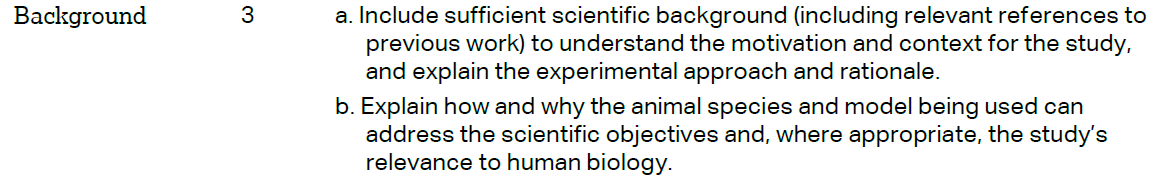 | | | Paragraphs 1-4  Paragraphs 4-5 |  |
| 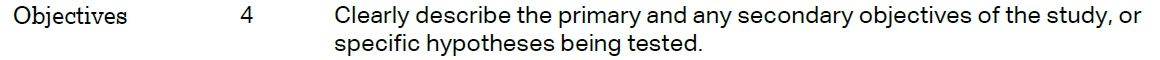 | | | Paragraph 5 |  |
| METHODS | | |  |  |
| 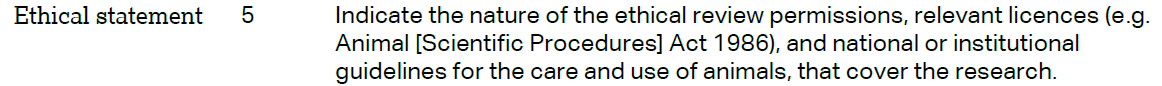 | | | Paragraph 2 |  |
| 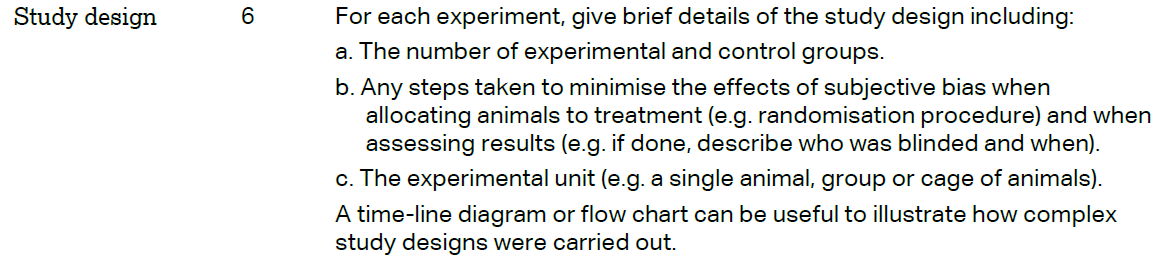 | | | Paragraphs 2-4 |  |
| 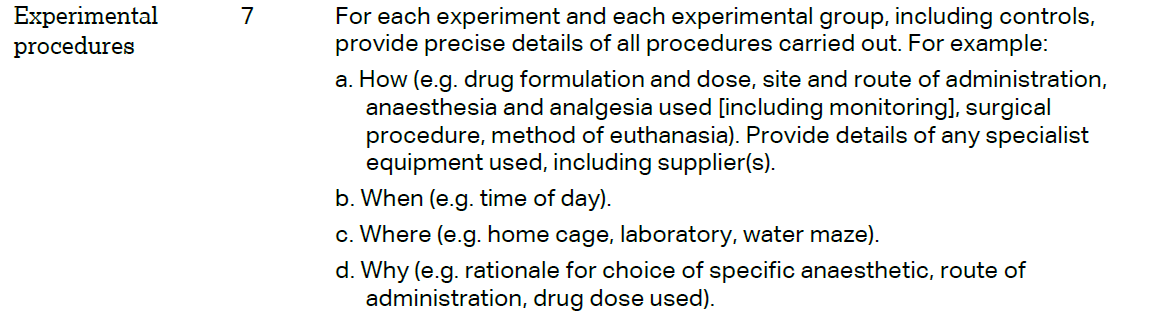 | | | Paragraphs 2-4 |  |
| 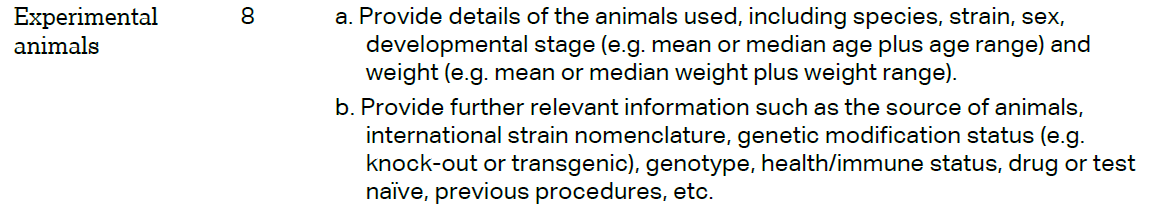 | | | Paragraph 2 |  |

The ARRIVE guidelines. Originally published in *PLoS Biology*, June 2010^1^

| 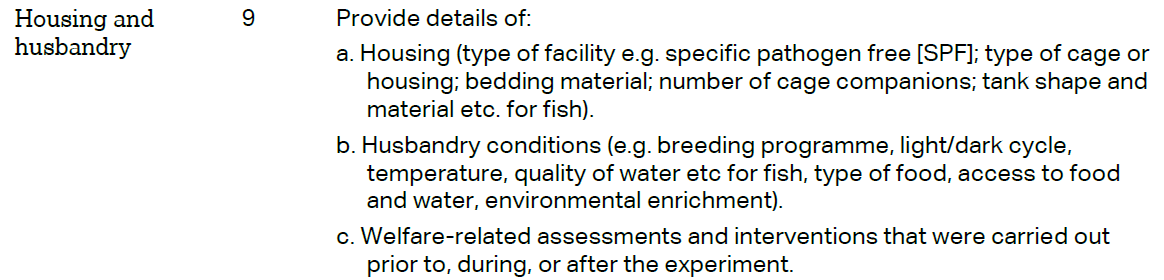 | Paragraph 2 | |
| --- | --- | --- |
| 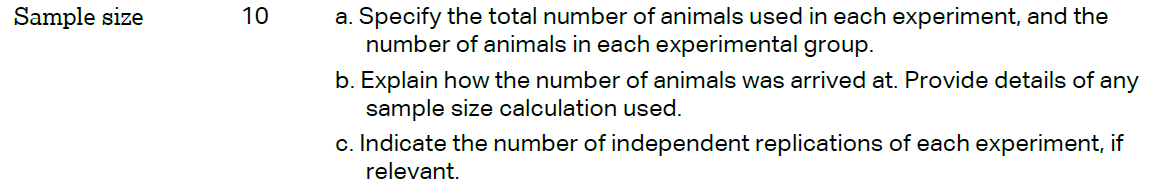 | Paragraph 3 | |
| 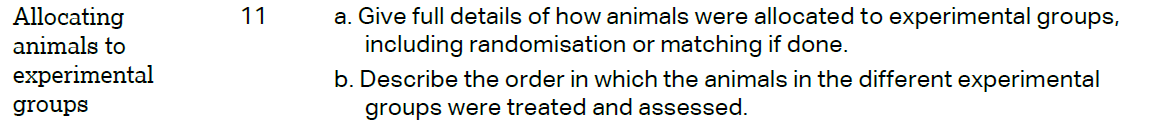 | Paragraphs 3-4 | |
| 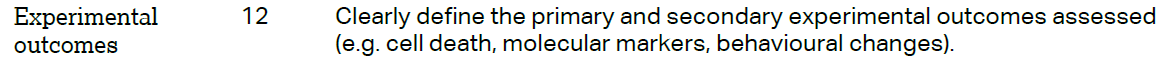 | Paragraph 5  Paragraph 14 | |
| 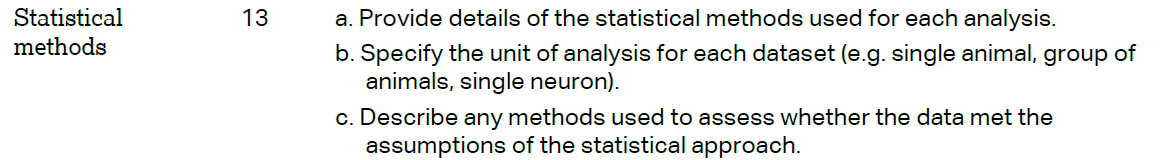 | Paragraphs 6-8 | |
| RESULTS |  | |
| 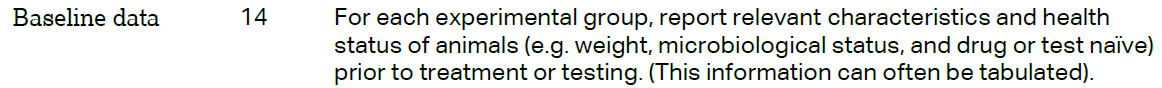 | Methods paragraph 2 | |
| 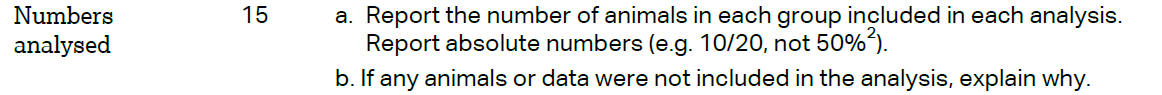 | Methods paragraphs 3-4 | |
| 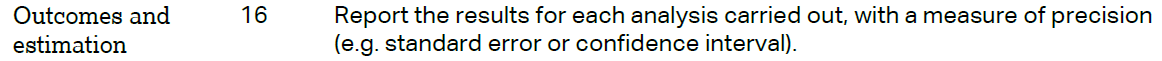 | Paragraphs 4-9  Tab. 1-2  Fig. 4-7 | |
| 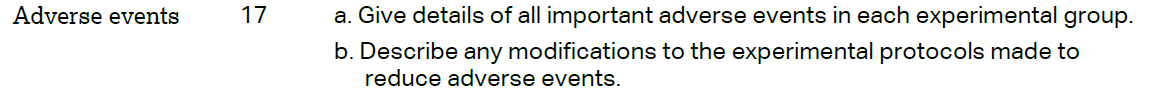 | No adverse events were observed during experiments. | |
| DISCUSSION |  | |
| 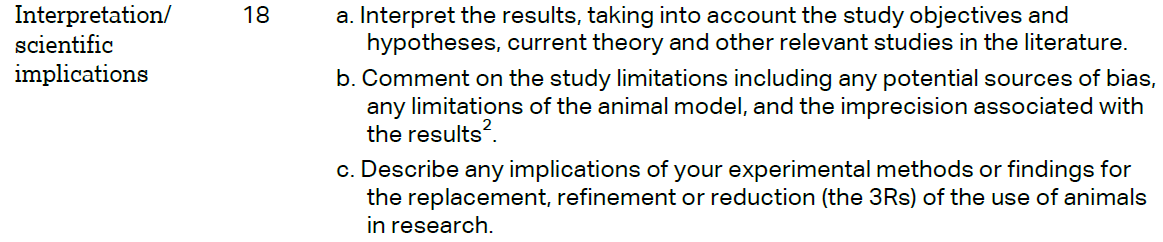 | Paragraphs 2-7 | |
| 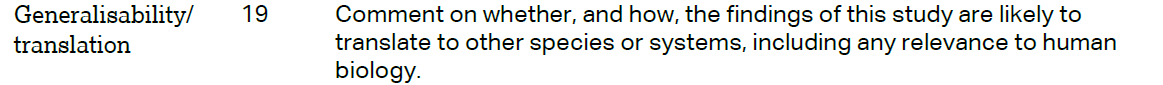 | paragraph 4 and 8 | |
| 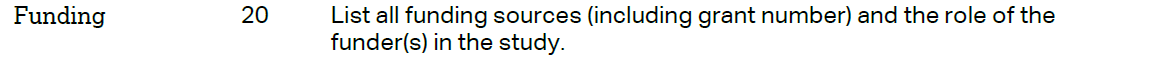 | | The Funding is not listed here. |


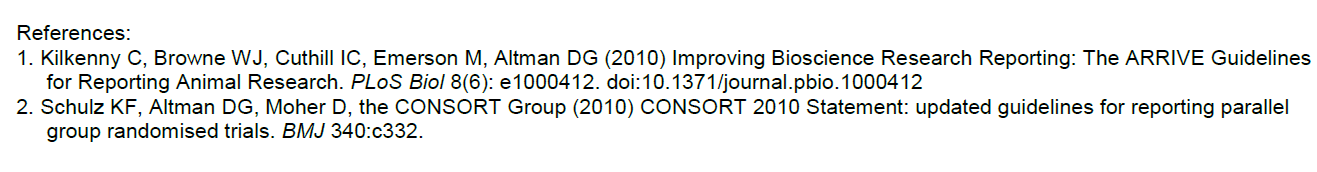

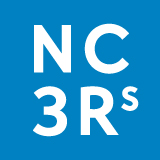

Supplement: S1 File — Completed “The ARRIVE Guidelines Checklist” for reporting animal data in this manuscript. (DOCX) [file pone.0128553.s001.docx]
